# Sustainable Polymer Composites Manufacturing through 3D Printing Technologies by Using Recycled Polymer and Filler

**DOI:** 10.3390/polym14183756

**Published:** 2022-09-08

**Authors:** Daniela Fico, Daniela Rizzo, Valentina De Carolis, Francesco Montagna, Carola Esposito Corcione

**Affiliations:** 1 Department of Engineering for Innovation, University of Salento, Edificio P, Campus Ecotekne, s.p. 6 Lecce-Monteroni, 73100 Lecce, Italy; 2 Department of Cultural Heritage, University of Salento, Via D. Birago 64, 73100 Lecce, Italy

**Keywords:** recycled polymer, 3D printing, sustainable polymer composites, circular economy, waste valorization

## Abstract

In the last years, the excessive use of plastic and other synthetic materials, that are generally difficult to dispose of, has caused growing ecological worries. These are contributing to redirecting the world’s attention to sustainable materials and a circular economy (CE) approach using recycling routes. In this work, bio-filaments for the Fused Filament Fabrication (FFF) 3D printing technique were produced from recycled polylactic acid (PLA) and artisanal ceramic waste by an extrusion process and fully characterized from a physical, thermal, and mechanical point of view. The data showed different morphological, thermal, rheological, and mechanical properties of the two produced filaments. Furthermore, the 3D objects produced from the 100% recycled PLA filament showed lower mechanical performance. However, the results have demonstrated that all the produced filaments can be used in a low-cost FFF commercial printer that has been modified with simple hand-made operations in order to produce 3D-printed models. The main objective of this work is to propose an example of easy and low-cost application of 3D printing that involves operations such as the reprocessing and the recyclability of materials, that are also not perfectly mechanically performing but can still provide environmental and economic benefits.

## 1. Introduction

Over time, the fast growth in material engineering has allowed the development of innovative materials, which are substituting conventional metals, alloys, and ceramics in many industrial fields [1,2,3]. In particular, composite materials are composed of two materials with different properties, whose combination results in a new material with increased performance. Among all the other composite materials, polymer composites and nanocomposites exhibit outstanding properties, suggesting their possible use in several industrial fields [4,5,6,7], such as aircraft and automobile industries [8,9], but also in the biomedical, architectural, textile, and design fields [10,11,12,13]. Unavoidably, the end life of all these objects causes a higher volume of plastic waste and has left the scientific world to study innovative ways of waste management and valorization [12,14,15,16]. Recycling approaches are thought to be preferable to both landfilling and incineration, due to the lack of recovery materials at the end of these processes [17,18,19]. According to academic opinion, severe legislation by the European Commission regarding the waste management of construction waste, vehicles, and electronic components are suggesting the composite-consumed industries investigate new and effective recycling approaches for industrial wastes [20,21]. Reusing is usually defined as the recycle of waste materials and often several procedures are necessary for waste recovery or valorization into products that can be utilized in different fields. The circular economy (CE) is also identified as a manufacturing method able to allow the remanufacturing, reprocessing, and recycling of materials [22,23]. A CE approach can not only help to reduce poisonous materials and waste but also create new products with good mechanical, thermal, and physical properties [13,24,25]. Among the different ways to recycle waste materials, Fused Filament Fabrication (FFF), an Additive Manufacturing (AM) technique, has recently been proposed for this purpose. In fact, the advent of AM has changed the way of producing 3D models. While in traditional manufacturing techniques the raw material should undertake numerous manufacturing steps, such as forming, machining, welding, etc., AM creates the components in a layer-based single-step manufacture process [26,27]. AM can be proposed for several application fields, even for space exploration purposes, such as fabricating different components of robots’ bodies [28] or for biomedical applications, such as the treatment of bone defects [15]. However, in order to maximize the properties of the 3D printed objects, it is very important to study the effects of voids and raster orientations on mechanical properties of notched additively manufactured PLA components [12], such as the fatigue life [29], the effective Young’s modulus [30], and the residual stress [31]. In addition, in order to obtain the best mechanical performances, it is necessary to define the optimum printing parameters for the different materials used [16,32,33]. As an example, Mohd Nazri Ahmad et al. (2022) [34] analyzed the optimal printing conditions using thermoplastic composites that had been reinforced with the use of oil palm fibers (0.4 mm layer thickness, flat (0 degree) of orientation, 50% infill density, and 10 mm/s printing speed) [34]. On the other hand, modal analysis of 3D printed structures is very important in order to improve the quality of the 3D printed models. The results obtained by Hieu Tri Nguyen et al. (2022) [35] showed that the adhesion type has the most important effect on the vibration response and parameters achieved from the modal analysis. Another important property that needs to be studied is the viscosity of the materials used in the FFF technique. In the work of Rossella Arrigo [36], the rheological behavior of several 3D-printable, commercially available poly(lactic acid)-based filaments was accurately characterized, with the aim to define an easy method to predict the FFF processability of thermoplastic materials [36]. Among the polymers used for FFF techniques, recently, several researchers have started to investigate the possibility of using biodegradable and/or recycled materials, such as a partial biodegradable blends prepared with polylactic acid (PLA) and polypropylene (PP) [37] or carbon black-carbonyl iron/polylactic acid composite filament [38]. In this framework, the aim of the present work is the design and characterization of innovative new filaments for the FFF technique composed of polylactic acid (PLA), ceramic scraps, and recycled PLA. The novelty of this manuscript is the investigation on the feasibility of sustainable polymer composites manufacturing through 3D printing technologies by using both recycled polymer and filler. The paper addresses an important issue regarding the raw material for the fabrication of functional materials, which is based on the use of waste to produce innovative materials. Lastly, for the first time, in this paper, the authors describe the modification of a commercially available FFF machine in order to use both biofilaments for the realization of a demonstrative simple 3D model, reaching further material and cost savings. To the best of our knowledge, this is the first example in literature of a modified FFF low-cost machine that can work with two different recycled filaments at the same time.

### Background of the Research

Medellin-Castillo and Zaragoza-Siqueiros (2019) [39] consider AM technologies suitable to meet the requirements of the economy, the environment, and the society. Moreover, in order for a product to be called “ideal”, it must be environmentally friendly, profitable for the producing company, and an improvement for society [39]. The terms sustainability and circular economy include these kinds of products. Therefore, scientific research conducted in recent years on FFF printing has concentrated on the development of innovative green filaments, produced from natural or recycled materials. The Additive Manufacturing (AM) research group of the Department of Engineering for Innovation of the University of Salento (Lecce, Italy) has been working for years on the recovery of different kinds of waste (domestic, industrial, handicraft), with the ultimate goal of giving it a second life, reducing its impact on the environment. From the recycling of common wastes, such as fruit or vegetable peels, fish bones, remnants of pizza, pasta, and bread, as well as excess flour, new methods have been developed for the production of bio-composite materials [24,25,40]. For example, the project “POIROT: Piattaforma domotica di Inertizzazione Rifiuti Organici Tracciabili” funded by “HORIZON 2020—PON I&C 2014–2020 FESR” (24-month duration), aimed at building an automated domestic equipment for the targeted disposal and recovery of organic waste through its transformation into stabilized/inert/valorized, identifiable, and reusable material. This research activity brought the development of thermoplastic polymeric bio-films composed using various waste flours derived from bakeries, pizzerias, or pasta factories [24]. The use of waste flour as a substitute for neat maize starch, usually used to produce green thermoplastic polymers, would avoid agricultural exploitation of neat starch production for industrial applications [24]. Among the different ways of recycling waste materials, the AM group has also demonstrated that 3D printing techniques allow an effective use of raw materials that can lead to low or zero waste manufacturing, reducing, in turn, carbon emissions not only in production but also in transport, since 3D models can be produced close to consumers [24,25]. The project “RE.CO.RD: REcycling strategies for the COastal sustainable waste management towards R&D Innovation”, financed under the Interreg VA Greece-Italy 2014–2020 Program, concerned the experimentation of new solutions for the recycling of plastic waste produced on the coasts of Apulia (Italy) and Greece, due to the high presence of touristic and economic activities. Part of the project involved the collection of PET bottles from the sea, which were properly treated and then used to extrude recycled PET filaments for FFF printing and for the production of new common objects, reducing the impact of plastics on the coastal environment [41]. From the need for economic recovery of small and medium-sized companies that emerged following the COVID-19 pandemic emergency, the recent research activities of the AM group have also focused on the recovery of processing waste from local companies and artisans in order to obtain design objects “made in Apulia”. For example, thanks to the use of FFF printing, PLA polymer matrix composite filaments have been developed, using scraps of Lecce stone as fillers [42]. This material is a calcarenite appreciated in Salento (Apulia, Italy) for its properties and known in Baroque architecture, but also it is a non-renewable resource and to be disposed. From these recycled filaments, building elements and furnishing objects were obtained [42]. Furthermore, the authors demonstrated the possibility to produce PLA/olive wood waste composites filaments for FFF [43], for the creation of objects of historical and traditional added value, and with economic savings and an ecological benefit quantified by Life Cycle Assessment (LCA) analysis [43]. As a continuation of our research activities, this work aims to be another example of a low-cost and easy recycling method, suitable for the recovery of ceramic and polymeric artisan production waste. In fact, FFF printing can be a valid alternative for artisans, as it offers them many advantages, such as the reduction in the use of raw materials, in the production costs, and the ability to create objects quickly without compromising their unique value.

## 2. Materials and Methods

This work is focused on the production of filaments from polymer and ceramic waste that can be used for FFF 3D printing technology, with the main purpose of reducing their impact on the environment, with ecological and economic benefits. In addition to the development of innovative green materials and the recycling of raw materials, another focus of the work is the modification of a commercially available printer for the use of the two biofilaments, achieving further material and cost savings. The morphological, thermal, and mechanical properties of the developed filaments were measured and compared to the ones of a commercial polymer filament and a virgin Polylactic acid (PLA) filament. The technical specifications of all the materials (subdivided for polymers and fillers) and the analytical techniques employed are given below.

### 2.1. Materials

All the tested samples were labelled as follows:

- PLA Ingeo 4043D: pellet (A), filament (AF) and 3D printed sample (A_3D);
- Recycled PLA Sunlu: waste (B), differentiated by color into transparent (Bt), white (Bw), blue (Bb), red (Br), and black (Bbk). Filament (BF) and 3D printed sample (B_3D);
- Ceramic waste: powder (C), filament (CF) and 3D printed sample (C_3D);
- Sunlu commercial filament (DF) and 3D printed sample (D_3D).

#### 2.1.1. Polymers

The Polylactic acid PLA Ingeo 4043D (labeled as A) used in the present work to produce the composite filament was purchased in pellet from the company NatureWorks LLC (Blair, NE, USA). This PLA is characterized by a density of 1.24 g cm^−3^ and a melt flow index (MFI) of 6 g/10 min at a temperature of 210 °C, according to the producer’s technical data sheet [13,44]. Specifically, this polymer has been used both alone to produce virgin filament (AF) and as a polymer matrix for the development of a composite filament containing ceramic scraps as a filler (labeled as CF). The pellet was first stored in an oven at 40 °C, then dimensionally reduced with the use of the Retsch ZM 100 Ultra Centrifugal Mill (Retsch GmbH, Haan, Germany) to a final size of 0.75 mm. The waste Polylactic acid (B) used for the production of the second filament (BF) was derived from unusable parts obtained from the 3D printing process (i.e., supports, test prints, failed objects, etc.) from PLA filaments of different colors (1.75 ± 0.02 mm diameter), purchased from the company Sunlu (Guangdong, China). According to the producer’s technical data sheet, it is characterized by a density of 1.24 g cm^−3^ and a melt flow index (MFI) of 7–9 g/10 min at a temperature of 190 °C. Sample B was first washed under tap water and dried at 60 °C, then dimensionally reduced to a final size of 0.75 mm, through the use of the SM100 cutting mill (Retsch GmbH, Haan, Germany) first and then the Retsch ZM 100 Ultra Centrifugal Mill (Retsch GmbH, Haan, Germany). 

#### 2.1.2. Filler/Scraps

The ceramic waste (labeled as C) added as fillers to the PLA polymer matrix (A) in percentages of 10% wt., for the production of the composite filament (labeled as CF) are artisan-derived and come from the ceramic workshop of the “*Fatto in bottega*” association (Brindisi, Italy). The raw material TFF-001505 was purchased from Colorobbia s.p.a. (Montelupo F.no, Italy) and consists of a classic white slurry derived from the mixture of several clays, supplied already filtered and degassed, ready to use. To obtain C filler powder, the waste was first washed under tap water and dried at 100 °C, then manually crushed with a hammer. Finally, the planetary ball mill of Ceramic Instruments company (Sassuolo, Italy) was used to reduce the ceramic waste into powder to a diameter of about 0.02–0.03 mm.

### 2.2. Production of Filaments for FFF Printing 

From the PLA pellets A (100% wt.), the filament AF was produced using a 3Devo Composer 450 Filament Maker (Utrecht, The Netherlands) with a single-screw extruder and using the parameters shown in Table 1. This filament and the commercial PLA filament (labeled as DF), purchased from the company Sunlu (Zhuhai, Guangdong, China) were used as references for morphological, thermal, and mechanical analyses. From the waste materials, two environmentally sustainable filaments were developed: the first from 100% wt. of recycled PLA waste (B) and named BF, and the second consisting of 90% wt. polymer matrix (A) and 10% wt. ceramic waste powder (C) as filler, labeled as CF. For the production of CF filament, PLA (0.75 mm) and ceramic powders (0.03 mm) were first manually mixed at room temperature and then put into the extrusion chamber. Extrusion of the filaments was always done using the 3Devo Composer 450 Filament Maker (Utrecht, The Netherlands) single-screw extruder and using the extrusion parameters are reported in Table 1.

### 2.3. Production of 3D Printed Samples for Bending Tests

The 3D printed samples were obtained by means of FFF technology by using the composite filaments achieved from waste materials (PLA and ceramic scraps). In accordance with the European Standard ISO178 (2014), bars with the dimensions of 80 mm × 10 mm × 4 mm were printed for bending tests, using the Creality CP-01 printer (Creality, London, UK) and setting the following operating conditions: extrusion temperature 200 °C, plate temperature 50 °C, printing speed 50 mm/s, infill 100%. The CAD model was created with Fusion 360 software (Autodesk, San Rafael, CA, USA), which was converted to an .STL file using Cura software (Ultimaker B.V., Utrecht, The Netherlands). All 3D printed samples produced from the waste filaments (B_3D and C_3D) were tested and compared with those obtained using DF and AF filaments (D_3D and A_3D), in order to compare their morphological, thermal, and mechanical properties. 

### 2.4. Printing a Representative 3D Model

**Figure 1:**
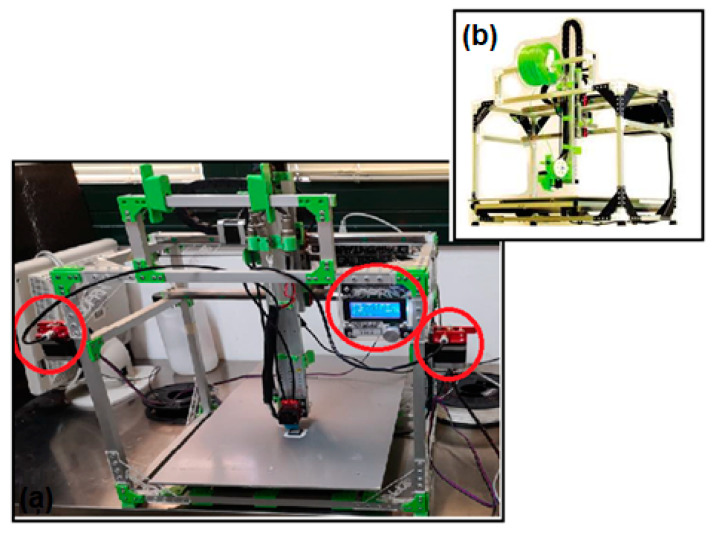
(**a**) 3DPRN LAB printed after modification of hardware and software components; (**b**) 3DPRN LAB commercial printed. The red circles are the modified parts of the 3D printer.

Finally, the filaments produced from the recycled polymer (BF) and waste ceramic (CF) were used to carry out a test print of a representative 3D object. To this end, the experimental activity was devoted to the modification of a commercial printer, model 3DPRN LAB (3DPRN company, Castiglione M.R., Italy), to make it suitable for the use of the two filaments (Figure 1). The red circles in Figure 1 represent the modified parts of the 3D printer. This allowed us to work with two different materials, and thus make two-color prints, or to use the filament with lower mechanical performance (BF) to create the removable supports of the object, with less waste of material and cost. Modifications to the printing press involved both hardware and software components. The following items were added for the hardware modification:

- 2 Redrex Aluminum extruders of Improved Bowden type with MK8 40-Tooth Tractor Gear with 2 different directions;
- 1 motor type 42BYGHW609 (12 V) to allow extrusion of a second filament;
- 5 drivers model A4988;
- 1 Hotend JINGERL BigTreeTech 2-in-1 out Hotend Dual Color Bowden Extruder 12 V PTF tube, dual filament input but with single groove in common.

Marlin firmware 2.0.9.1 was downloaded from the website http://marlinfw.org/ (accessed on 12 August 2022) [45] and modified with the open-source Arduino IDE 1.8.15 software, to configure the dual extruder during the printing process. The modified firmware was uploaded to the electronic board Megatronic V3.0 of the printer. The CAD model was created with Fusion 360 software (Autodesk, San Rafael, CA, USA), which was converted to an .STL file using Cura software (Ultimaker B.V., Utrecht, The Netherlands).

### 2.5. Methods

#### 2.5.1. Morphological Analysis

Morphological analysis was performed on all the filaments with a scanning electron microscope (SEM, Zeiss E Evo 40, Oberkochen, Germany), with the aim of studying the uniformity or dissimilarity of surfaces, following the recycling of material and the addition of ceramic filler. Magnification images of the 3D printed bar sections were obtained using the Dino-Lite Digital Microscope instrument (AnMo Electronics Corporation, New Taipei City, Taiwan), to understand the morphology and adhesion of the different layers deposited.

#### 2.5.2. X-ray Diffraction Analysis (XRD)

XRD analysis (Rigaku Ultima+, Tokyo, Japan) was performed with CuKα radiation (λ = 1.5418 Å) in the step scan mode recorded in the 2θ range of 10°–60°, with a step size of 0.02° and a step duration of 0.5 s. XRD analyses were performed on both raw materials and filaments.

#### 2.5.3. Differential Scanning Calorimetry Analysis (DSC)

DSC analysis (Mettler Toledo DSC1 StareSystem) was performed to evaluate the influence of the recycling process on PLA and the effect of the addition of ceramic filler in the polymer matrix on glass transition temperature (T_g_) and melt temperature (T_m_). The analyses were carried out using a temperature range of 25 to 200 °C (10 °C/min) and were performed on both raw materials, filaments, and 3D printed bars. In addition, the percentage of crystallinity χc (%) of each filament produced was calculated according to Equation (1) [43,46]:

χc = (H_m_ − H_c_)/(H_∞_ [1 − (%weightfiller/100)]) (1)

where H_m_ is the enthalpy of melting, H_c_ is the enthalpy resulting from crystallization, H_∞_ is the enthalpy of melting of fully crystallized PLA, assumed equal to 93.7 J/g, and % weight filler is the content of filler C [46]. 

#### 2.5.4. Rheological Analysis

The Rheometrics Ares model rheometer (TA Instruments, New Castle, DE, USA) was used for rheological analysis of raw materials and mixtures. Constant speed tests were performed at 200 °C, with a parallel plate geometry, varying the shear rate from 0.01 to 35 s^−1^. A temperature of 200 °C was chosen because it is the 3D printing process temperature. The analyses were performed at least five times to verify the accuracy of the results. 

#### 2.5.5. Mechanical Characterization of 3D Printed Samples

According to the ISO178(2014) standard, flexural tests were performed on all 3D printed bars using a Lloyd LR5K dynamometer (Lloyd Instruments Ltd., Bognor Regis, UK), with a test speed of 2 mm/min and a specimen support spacing of 64 mm. Five replications were performed for each specimen.

## 3. Results and Discussion

### 3.1. Characterization of Neat Materials

**Figure 2:**
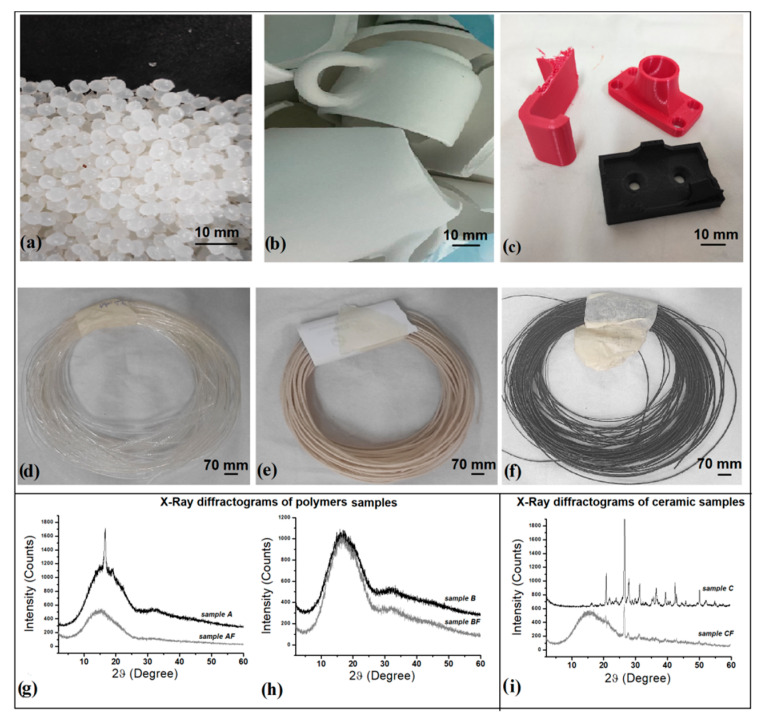
Images of the raw materials used in the production of composite filaments: (**a**) PLA Ingeo 4043D (A); (**b**) ceramic waste (C); (**c**) objects printed in PLA Sunlu (B). Images of filaments: (**d**) AF filament; (**e**) CF filament; (**f**) BF filament. X-Ray diffractograms of raw materials (polymers and filler) and of innovative green filaments developed in the work: (**g**) comparison of A and between the respective filament produced AF; (**h**) comparison of B and between the respective filament produced BF; (**i**) comparison of ceramic waste (C) and between the respective filament produced CF.

All raw materials (Figure 2a–c) used for the production of the biofilament (Figure 2d–f) were characterized from a structural and thermal point of view by XRD and DSC analysis.

#### 3.1.1. Polymers

**Figure 3:**
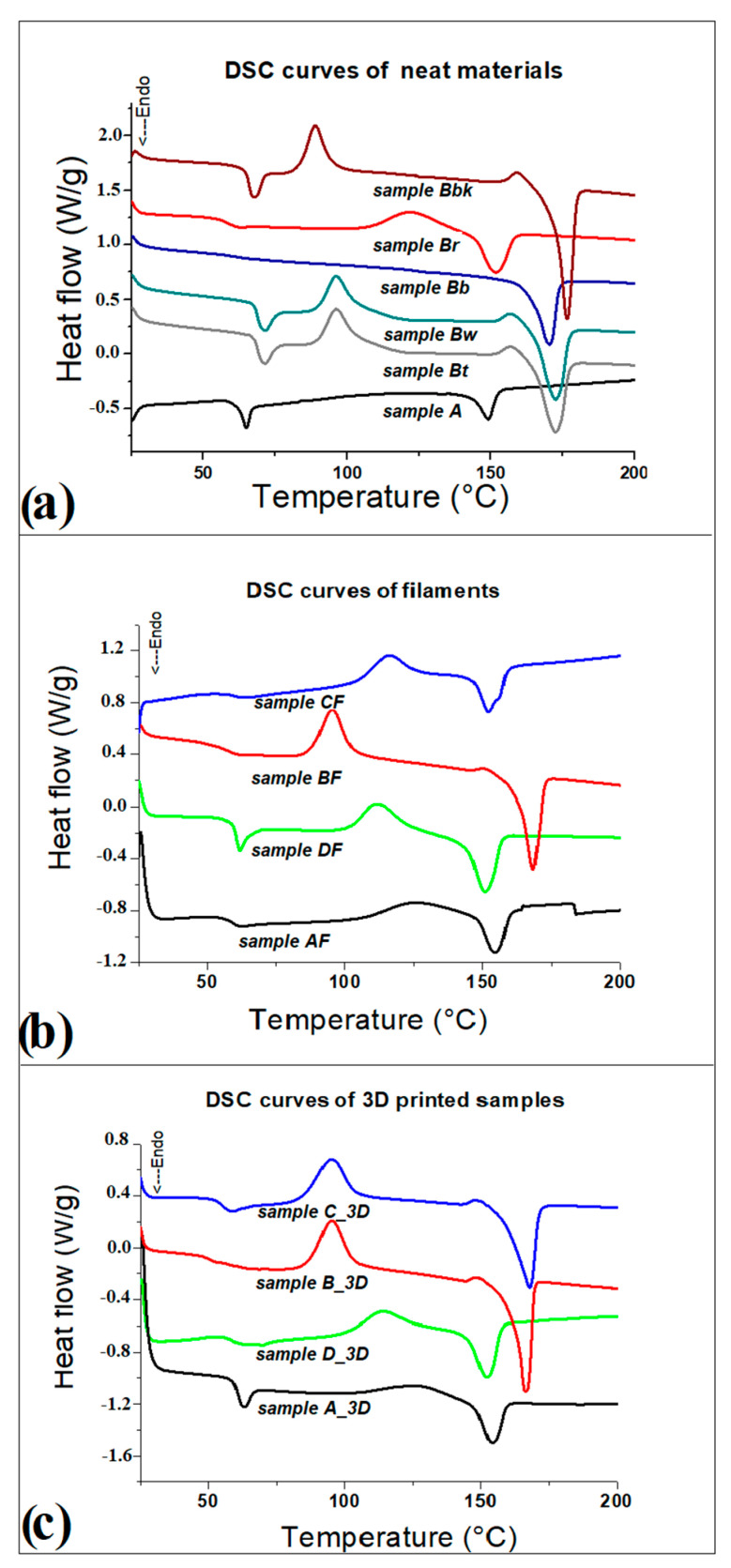
DSC thermograms of the samples: (**a**) polymeric neat materials; (**b**) filaments; (**c**) 3D printed samples.

The X-ray diffractogram of the A sample (Figure 2g) shows a diffuse diffraction band from which a more defined peak emerges at 2θ (°) = 16.7°, assigned to the crystalline plane (110), according to the literature [13,43]. The X-ray diffractogram of the B sample (Figure 2g) shows a diffuse diffraction band at about 2θ (°) = 16.7° from which two more defined diffraction peaks emerge at 9.38° and 14.54°. The processing of the material and subsequent annealing of PLA generated the formation of crystals, which partially increase the degree of crystallinity compared to virgin PLA [47,48]. DSC thermograms obtained from the analysis of different polymers used as raw materials are shown in Figure 3a. The DSC curves of the of the differentially colored recycled PLA (Sanlu brand) appear different from each other. This is probably due to the additives and dyes used in the production of the commercial filaments, which are not specified in the data sheets of the producer. The glass transition temperatures (T_g_) of all materials were measured with STARe System software (Mettler Toledo, Milan, Italy), using the inflection point as the measurement point (Table 2): the T_g_ coincides with the point at which the second derivative is zero. The measured T_g_ for A sample is 60.61 °C, values similar to those reported in the literature [13,43,49]. The Br and Bb samples show T_g_ similar to this value, while a higher T_g_ was measured for Bt, Bw and Bbk, with values between 65.82 °C and 68.76 °C. Some thermograms show the absence of cold crystallization: for example, A and Bb samples. In contrast, the other polymers show crystallization temperatures (T_c_) between 88.74 °C and 121.66 °C (Table 2). Melting temperature (T_m_) values ranging between 151.74 °C and 176.82 °C. Overall, the results indicate an increase in crystallinity of all B samples derived from recycled PLA, evident from the percentage of crystallinity χc (%), calculated according to the Formula (1) and shown for each sample in Table 2. The data obtained from DSC analysis agree with those from XRD analysis: the presence of more diffraction peaks for B sample, compared to A sample, is evident. These samples indeed underwent additional heat treatment due to 3D printing, and this process changes the initial thermal properties, as expected [47,48]. 

#### 3.1.2. Filler/Scraps

XRD analysis was performed on the waste ceramic powder used as filler added to the polymer matrix to create the composite filament. The X-ray diffractogram of sample C (Figure 2i) shows a series of main peaks 2θ (°) that can be associated with the different minerals original of the row ceramic material, respectively at: 20.79° (Feldspar, KAlSi_3_O_8_), 26.59° (Quartz, SiO_2_), 27.80° (Dolomite, CaMgCO_3_), 31.30°, 36.38° (Calcite, CaCO_3_), 39.52° (Calcite, CaCO_3_), 42.43°, 42.79°, 50.05° (Quartz, SiO_2_), 59.97° (Almandine, Fe_3_Al_2_(SiO_4_)_3_) [50,51]. The predominant peaks are related to silicon and calcium as major elements.

### 3.2. Morphological, Structural, and Thermal Characterization of Composite Filaments

**Figure 4:**
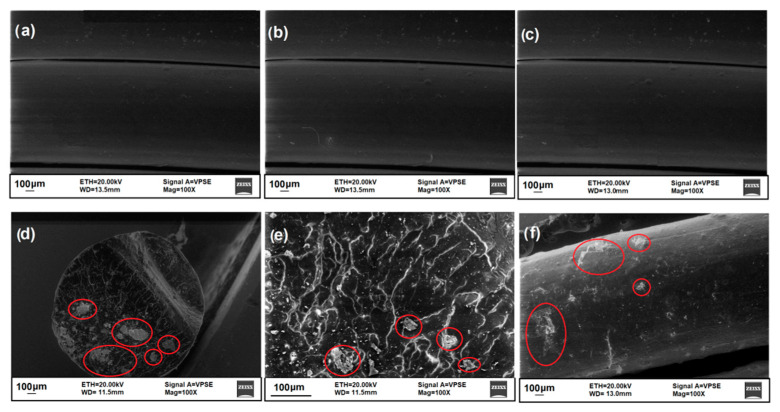
SEM images of: (**a**) DF filament (100X); (**b**) AF filament (100X); (**c**) BF filament (100X); (**d**) section of filament CF (100X); (**e**) section of filament CF (500X); (**f**) CF filament (100X).

SEM analyses were performed on the filament section to observe the homogeneity/heterogeneity of the structure and on the outer surfaces to study the surface roughness. Differences between the filaments at the outer surface level emerge from the results (Figure 4): the outer surfaces of the DF (Figure 4a), AF (Figure 4b), and BF (Figure 4c) filaments are perfectly smooth, with no obvious discontinuities; the addition of C particles creates an irregular filament surface, and some discontinuities are observed (Figure 4f). In addition, while the sections of DF, AF, and BF filaments are homogeneous, the addition of C filler does not occur uniformly in the polymer matrix: ceramic particles can be distinguished in the section of the composite CF (Figure 4d,e) and in some cases form large aggregates (highlighted in the SEM images with red marks).

XRD spectra acquired on the filaments detect a significant decrease of the intensity of diffraction peaks (Figure 2g–i). The polymer filaments (AF, BF) show an amorphous band (Figure 2g,h), while in the CF filament, few ceramic powder diffraction peaks remain above the main amorphous band (Figure 2i). Main peaks are located at 2θ (°) equal to 26.35°, 27.67°, and 31.07°, respectively. More precise information on the crystallinity of extruded filaments was acquired from DSC analysis. Figure 3b shows the DSC thermograms obtained from the analysis of the different filaments produced, compared with the commercial filament DF. Moreover, by calculating melting and crystallization enthalpies, it was possible to determine the degree of crystallinity reported in Table 3. Overall, a decrease of the T_g_ is observed in all extruded filaments, compared with the commercial filament DF or raw materials. The extrusion process carried out by 3D Evo machine at different temperatures results in molecular reorganization, with a consequent change in properties compared to the raw materials [52]. However, the major differences due to this process are found in the crystallization (H_c_) and melting (H_m_) temperatures (Table 3). For example, the AF compared with the pellet A indeed possesses an obvious crystallization peak at 124.39 °C, which is absent in the DSC curve of the pellet, and the melting temperature undergoes an increase from 153.57 °C to 154.68 °C after extrusion. In fact, the degree of crystallinity χc (%) underwent an increase from 0.17 to 0.30 %. The BF and CF filaments produced from waste materials (recycled polymers and ceramic waste) show a similar trend with a more noticeable increase in crystallinity, compared with virgin PLA. This, in the case of polymers, may be due to the use of recycled plastic material, which due to the shear stresses, temperature and oxygen conditions that occur during extrusion cause degradation, with an increase in material crystallinity and melting temperature [53]. The addition of ceramic filler, on the other hand, results in an increase in the crystallinity of the filament compared to virgin PLA due to the increased stiffness of the polymer chains. However, between the two filaments, the CF still possesses a lower χc (%) than the BF filament.

**Figure 5:**
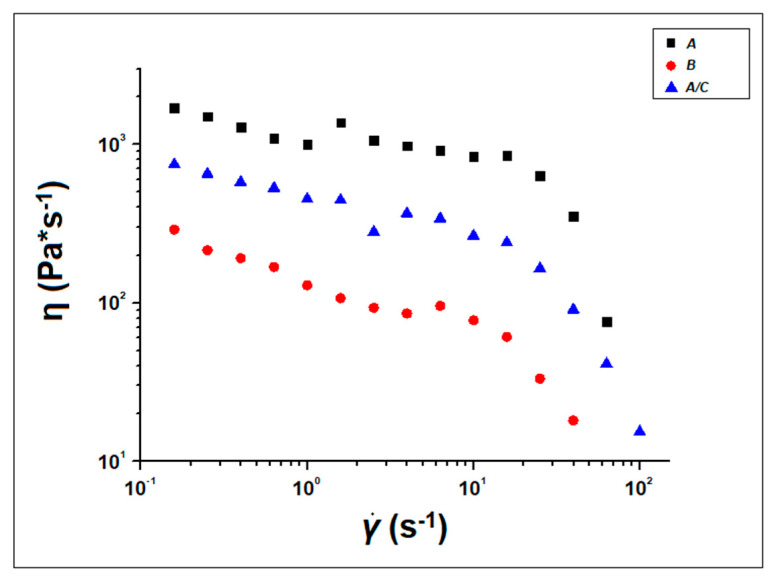
Rheological curves of A, B, and A/C blend.

To obtain information on the printability of the filaments developed from the waste materials, rheological measurements were carried out (Figure 5). Specifically, the change in viscosity was recorded by setting a temperature of 200 °C and a shear rate ranging between 10 s^−1^ and 100 s^−1^ to simulate the conditions used in FFF printing [42,43]. The results show that viscosity decreases as shear rate increases, and both polymers (A, B) and the A and C powder mixture (A/C) have a pseudoplastic behavior [42,54]. The rheological curves appear almost superimposable: a slight decrease in the viscosity of the B and of the mixture with ceramic fillers is observed, making both materials suitable for 3D printing process.

### 3.3. Thermal, Mechanical, and Morphological Characterization of 3D Printed Bars

DSC analysis performed on the samples printed by FFF showed no significant changes between the extruded AF filament and the relative 3D bar A_3D (Table 4). A decrease of the glass transition temperature (T_g_) is observed in all other 3D samples. From the enthalpy changes (ΔH_c_ and ΔH_m_), there is also evidence of an increase in the crystallinity of the materials (χc), due to thermal reprocessing of the printing process [42,47]. Among these, the PLA and waste ceramic composite (C_3D) shows the highest value of χc (%).

**Figure 6:**
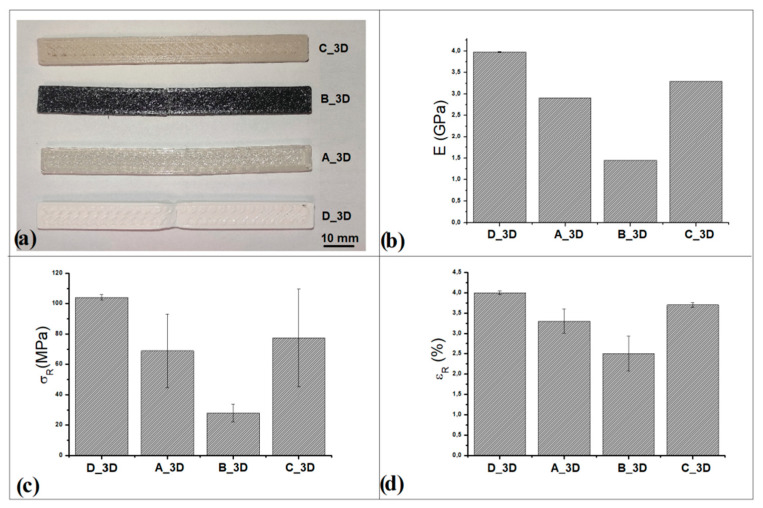
(**a**) 3D bars: D_3D, A_3D, B_3D and C_3D. Mechanical flexural properties: (**b**) flexural modulus of elasticity (GPa), (**c**) flexural stress (MPa), (**d**) flexural strain (%).

The mechanical performance of the developed filaments was evaluated by subjecting the 3D printed bars to bending tests, following the ISO178 (2014) standard. PLA is a widely used polymer in FFF printing technologies since it has excellent strength and hardness properties in addition to eco-friendliness and non-toxicity [55]. However, several authors highlight the influence of some printing process variables (raster angle, layer height, and raster width) on the bending properties of FFF-printed polylactic acid [13,55,56,57]. For example, Liu et al. (2019) [56] in their study on virgin PLA and several derived composites achieved high mechanical strength in their experiments using +45°/−45° raster angles on both virgin PLA and PLA matrix composites [56]. Therefore, based on work reported in the literature [56,57] and our recent research [13,43], this orientation was selected to compare all 3D printed samples. The results obtained from the mechanical tests (Figure 6) show values of the flexural properties of D_3D and A_3D bars comparable with the scientific literature [43,58]. However, heating of the PLA due to the printing process results in a decay of the ulterior mechanical properties in the B_3D sample [52,54,59], which results in a reduction of the flexural modulus of elasticity (GPa) by half. The largest decrease is observed for σ_R_ (MPa), while the decrease related to ε_R_ (%) is smaller. Cruz Sanchez et al. (2017) showed that the costs of commercial PLA filaments are up to 200 times higher than those of raw plastics [59]. Obtaining filaments from recycled polymers with mechanical performance similar to virgin materials would also significantly contribute to reducing the cost of 3D printing. The data obtained in this work on the B_3D sample are not impressive, but the use of filament in specific 3D printing applications would still provide economic and raw material savings, as demonstrated by the authors in the next section. Overall, studies have shown that by adding metal or ceramic powders to virgin PLA, the tensile and flexural modulus, strength, and strain at break are unchanged or even increased compared with those of virgin PLA, using different raster angles [13,50,56]. In our work, the mechanical performance related to the ceramic composite bar C_3D is similar to the properties of virgin PLA, indicating the good performance of composite CF.

**Figure 7:**
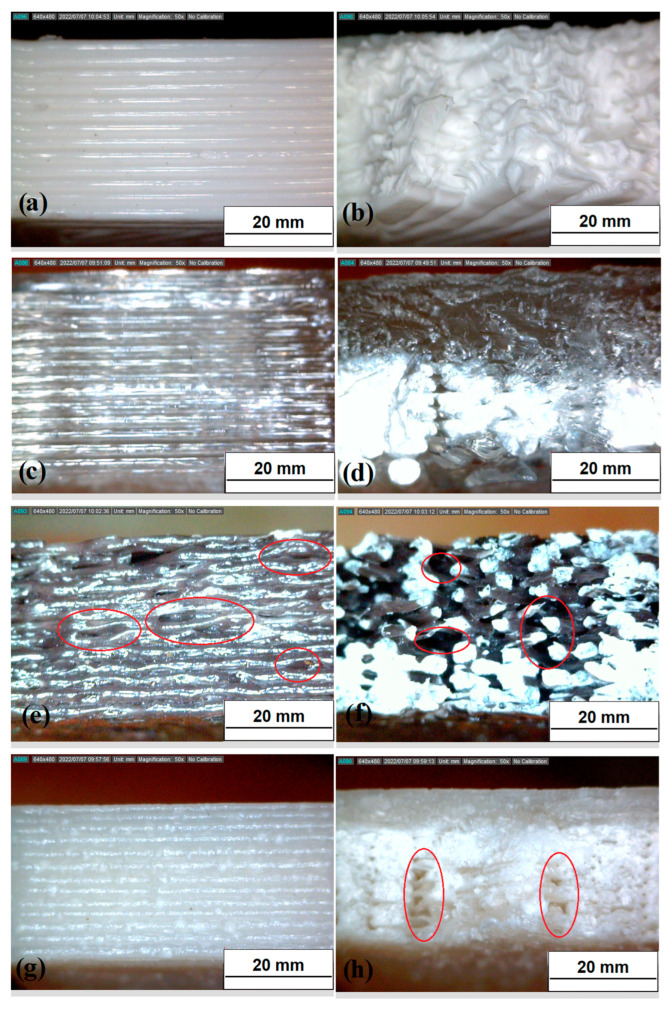
Magnified images (50×) of the 3D bars: (**a**) printing surface of D_3D specimen; (**b**) breaking surface after bending test of D_3D specimen; (**c**) printing surface of A_3D specimen; (**d**) breaking surface after bending test of A_3D specimen; (**e**) printing surface of B_3D specimen; (**f**) breaking surface after bending test of B_3D specimen; (**g**) printing surface of C_3D specimen; (**h**) breaking surface after bending test of C_3D specimen.

Finally, to further understand the properties of green innovative developed filaments an analysis of layers and of the fracture morphology for 3D bars was performed. Optical images are showed in Figure 7. The images evidence a good adhesion between 3D layers in the polymeric samples D_3D (Figure 7a,b) and A_3D (Figure 7c,d), and in the composite sample C_3D (Figure 7g,h). The addition of ceramic filler causes the formation of particle aggregates in the polymer matrix in some areas of the filament, as seen from SEM images of the filaments (Figure 4d,e); however, this does not cause adhesion problems between the print layers (Figure 7g). Each 3D printed layer is perfectly aligned and does not exhibit any deformation phenomena, such as swelling, warping, detachment, or shifting. However, from observation of the fracture surface (Figure 7h), only a few air gaps are observed. This porosity does not inhibit the mechanical performance of the composite material. In contrast, the B_3D sample (Figure 7e,f) shows poor adhesion of the layers, air gaps, and poor overall compaction in some areas. The main defects are highlighted in Figure 7e,f by the red circles. The morphology of the structure is defective, which explains the lower mechanical performance of recycled PLA. In fact, the thermomechanical degradation undergone by this material involves change in molecular weight and therefore lower breaking strength [59,60], and this process is more evident as the number of processing cycles increases [47,54,58,59,60]. Misalignment of 3D printed layers and deformation of surfaces is also evident, indicating fragile behavior of the recycling PLA.

The reduced flexural properties of recycled PLA, however, make it suitable for a variety of applications, such as in the non-structural field, or in sustainable applications that involve less waste of raw materials, as better described in the next section.

### 3.4. Example of Sustainable Application

In this section, the authors report an example of a sustainable application of 3D printing FFF through the engineering modification of a commercial printer 3DPRN LAB and the use of recycled materials. The reuse of materials that are not easily disposable such as ceramics, or biodegradable but still take several months to degrade and have purchase costs, such as Polylactic acid, provides environmental and economic benefits. Scientific research, especially that based on Life Cycle Assessment (LCA), shows significant economic savings from the addition of industrial waste fillers, compared to virgin Polylactic acid (PLA), and a transition to the circular economy [43,61]. The ability to recycle PLA, the polymer most used in FFF printing, through a simple hand-made process obtaining a filament that can be reused again for the same print also allows for 100 percent material recovery at zero cost. Recycled PLA filament, although having less than excellent mechanical properties, can in fact be used to produce printing supports, which are then removed in the finishing stages of the object after printing or for the construction of secondary parts, such as filling of the objects, which need not be visible from the outside.

**Figure 8:**
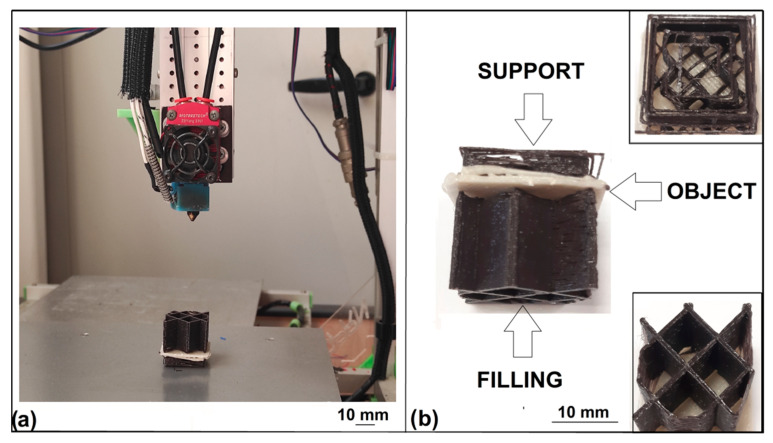
(**a**) Representative sample printed with the 3DPRN LAB printer; (**b**) 3D printed representative sample components and details.

In the present work, a representative example of a 3D model with a simple geometry (Figure 8a) was produced through the use of the modified 3DPRN LAB printer and the two developed filaments BF and CF. A square was selected as a representative sample, printed using the good quality CF filament (Figure 8b), while the supports (usually necessary for printing objects of complex geometry) and the inner filling (usually not visible but necessary to impart structural properties to the object), were printed using the BF filament, which has lower mechanical properties (Figure 8b). The operating parameters used for printing using the two filaments were: nozzle 1 mm, extrusion temperature 200 °C, plate temperature 50 °C, printing speed 40 mm/s, movement speed 150 mm/s, retraction distance 5 mm, layer height 0.32 mm, infill of object 100%, infill of support 20%, and filler infill 2%.

## 4. Conclusions

Currently, considerable attention is being devoted to the development of sustainable materials and technologies in order to limit the environmental matters generated by different kind of waste and, in particular, plastic materials. Most of the wastes are commonly managed by incineration and landfilling techniques; however, both approaches are not completely sustainable. A possible eco-friendly solution for the widespread production of waste could be the development of innovative recycling strategies. Among these, Fused Filament Fabrication (FFF), an Additive Manufacturing (AM) technique, has recently been proposed for this purpose, due to its versatility and to its applicability in the sustainability field. In fact, through recent years, many studies have been aimed at improving the sustainability of the commercial materials used in 3D printing, causing the development of innovative biodegradable and green composite filaments.

In this paper, a possible waste recycling strategy using the FFF technique is proposed. Polylactic acid (PLA) was recycled from unusable and accumulated 3D printing parts and then used to produce two composite biofilaments for FFF, by adding waste ceramics as filler to pure PLA matrix. These ceramics derived from broken pieces of cups, vases, and furnishings produced by some Apulian artisans. A complete physical, thermal, and mechanical characterization of the new filaments was made. The thermal and rheological characterization evidenced that the presence of the ceramic scraps does not substantially change the properties of the neat polymer. Furthermore, the crystallinity of the ceramic filament (10% wt.) increases compared to pure PLA and morphological analysis shows that the dispersion of ceramic particles is not uniform in the polymer matrix. Despite this, the mechanical properties of the 3D printed objects are good, associated with adequate adhesion between layers. In contrast, the final performances of the recycled PLA are decreased. Printed layers do not bond well, and numerous gaps and surface defects are observed. Despite this, both filaments produced were suitable to be used in a low-cost 3D printing machine, in order to build a simple 3D printed model. Therefore, throughout this work, we demonstrated the suitability of the proposed approach to successfully recycle different kinds of artisanal waste, with increased ecological and economic benefits. Our future research is moving toward optimizing the biofilaments produced, such as through the addition of plasticizers that can increase homogeneity of the ceramic dispersion in the matrix, and stabilizing thermal agents that make the reused PLA stable and with improved properties. Currently, the biofilaments developed can find applications in the craft and design sectors, and can be used, for example, by 3D printing makers, designers, artists, and artisans through the use of a simple and cost-effective technology that supports a circular economy.

## Figures and Tables

**Table 1:** Extrusion process parameters for filaments: filament 100% wt. PLA Ingeo 4043D (AF), filament 100% wt. PLA Sunlu waste (BF), filament 90% wt. PLA Ingeo 4043D and 10% wt. ceramic waste powder (CF).

Extrusion Process Parameters	AF	BF	CF
Screw speed (rpm)	3.5	4.0	3.5
Feed zone temperature (°C)	170	195	200
Compression zone temperature (°C)	185	190	190
Metering zone temperature (°C)	190	190	210
Die temperature (°C)	200	200	220

**Table 2:** Thermal properties of polymers and percentage of crystallinity χc (%).

Label	Weight Composition (% wt.)	T_g_ (°C)	T_c_ (°C)	T_m_ (°C)	χc (%)
A	100 PLA Ingeo 4043D	60.03	/	153.57	0.17
Bt	100 PLA Sunlu waste trasparent	68.76	96.25	172.42	0.62
Bw	100 PLA Sunlu waste white	68.27	95.95	172.75	0.41
Bb	100 PLA Sunlu waste blue	60.15	/	170.25	0.27
Br	100 PLA Sunlu waste red	58.95	121.66	151.74	0.40
Bbk	100 PLA Sunlu waste black	65.82	88.74	176.82	0.71

**Table 3:** Thermal properties of filaments and percentage of crystallinity χc (%).

Label	Weight Composition (% wt.)	T_g_ (°C)	T_c_ (°C)	T_m_ (°C)	χc (%)
AF	100 PLA Ingeo 4043D	59.19	124.39	154.68	0.30
BF	100 PLA Sunlu waste	56.64	95.52	168.09	0.54
CF	90 PLA Ingeo 4043D and 10 Ceramic waste powder	59.40	116.11	152.24	0.42
DF	100 commercial PLA Sunlu	60.61	111.67	150.83	0.08

**Table 4:** Thermal properties of 3D bars printed and percentage of crystallinity χc (%).

Label	Weight Composition (% wt.)	T_g_ (°C)	T_c_ (°C)	T_m_ (°C)	χc (%)
A_3D	100 PLA Ingeo 4043D	60.82	126.99	153.95	0.29
B_3D	100 PLA Sunlu waste	50.47	95.17	166.10	0.61
C_3D	90 PLA Ingeo 4043D and 10 Ceramic waste powder	55.11	95.16	167.78	0.66
D_3D	100 commercial PLA Sunlu	58.84	113.53	152.14	0.41

## Data Availability

Not applicable.
